# Synthesis, Photoluminescence and Electrical Study of Pyrazolone-Based Azomethine Ligand Zn(II) Complexes

**DOI:** 10.3390/ma13245698

**Published:** 2020-12-14

**Authors:** Alexey Gusev, Elena Braga, Andrey Tyutyunik, Vladimir Gurchenko, Maria Berezovskaya, Mariya Kryukova, Mikhail Kiskin, Wolfgang Linert

**Affiliations:** 1 General and Physical Chemistry Department, Crimean Federal University, 295007 Simferopol, Russia; galex0330@gmail.com (A.G.); braga.yelena@ya.ru (E.B.); real-warez@mail.ru (A.T.); gurchenko_v@mail.ru (V.G.); tnupt@mail.ru (M.B.); 2 Institute of Chemistry, Saint Petersburg State University, 199034 Saint Petersburg, Russia; mary_kryukova@mail.ru; 3 N.S. Kurnakov Institute of General and Inorganic Chemistry, Russian Academy of Sciences, 119991 Moscow, Russia; m_kiskin@mail.ruKiskin; 4 Institute of Applied Synthetic Chemistry, Vienna University of Technology, A-1060 Vienna, Austria

**Keywords:** pyrazolone-based ligands, Schiff bases, metal complexes, luminescence

## Abstract

New luminescent zinc complexes were obtained by reaction of pyrazolone-based azomethine ligands with Zn(CH_3_COO)_2_·2H_2_O. Complexes fully characterized by elemental analysis, FTIR, ES–MS, NMR, and single crystal X-ray analysis. Title complexes in the solid state demonstrate tunable luminescence from blue to orange by varying of substituents on the aromatic ring. Quantum yields are in the 0.03 to 0.49 range. TGA data shows that obtained complexes demonstrate high thermal stability and can be used as electroluminescent materials. The electrical properties of the complexes under study were considered in the ITO-Zncomplex-Al “sandwich” structure.

## 1. Introduction

Coordination compounds demonstrate sustained interest as the platform of functional materials over the past several decades. The structure and related properties of such coordination compounds widely vary due to fine-tuning of the structure of organic ligands, types of metals, the ratio of components, reaction conditions, etc. Moreover, specific and often unique optical properties, identified interest to title compounds for the potential applications of title complexes as efficient dye in dye sensitized solar cells and imaging agents in biological systems [1,2,3,4,5,6]. Luminescent complexes may be suitable for use as the emitting agents for the emissive layers in OLED (organic light-emitting diodes), and other organic electronic devices. In both cases, the key factor determining the practical use of such complexes is their intense luminescence in combination with a low price [7,8,9]. Among the promising optical materials, zinc complexes have been especially important because of the simplicity in their synthesis procedures, wide range of emission maximum position and low cost of zinc in comparison with platinum metals and f-elements. Extensive research work in recent decades has led to the preparation of a number of new zinc complexes containing organic ligands [10,11,12,13,14,15,16,17,18,19,20,21,22,23,24], which have been used as emitters and electron transporters in OLED research. The molecular design and synthesis of new organic ligands are among the main approaches for the construction of zinc complexes with controllable luminescent properties. Among the large number of zinc complexes azomethine ligands proved to be excellent candidates for the development of new luminescent material due to the ease of introduction and targeted variation of type and position of substituents [16,17,18,19,20,21].

**Scheme 1:**
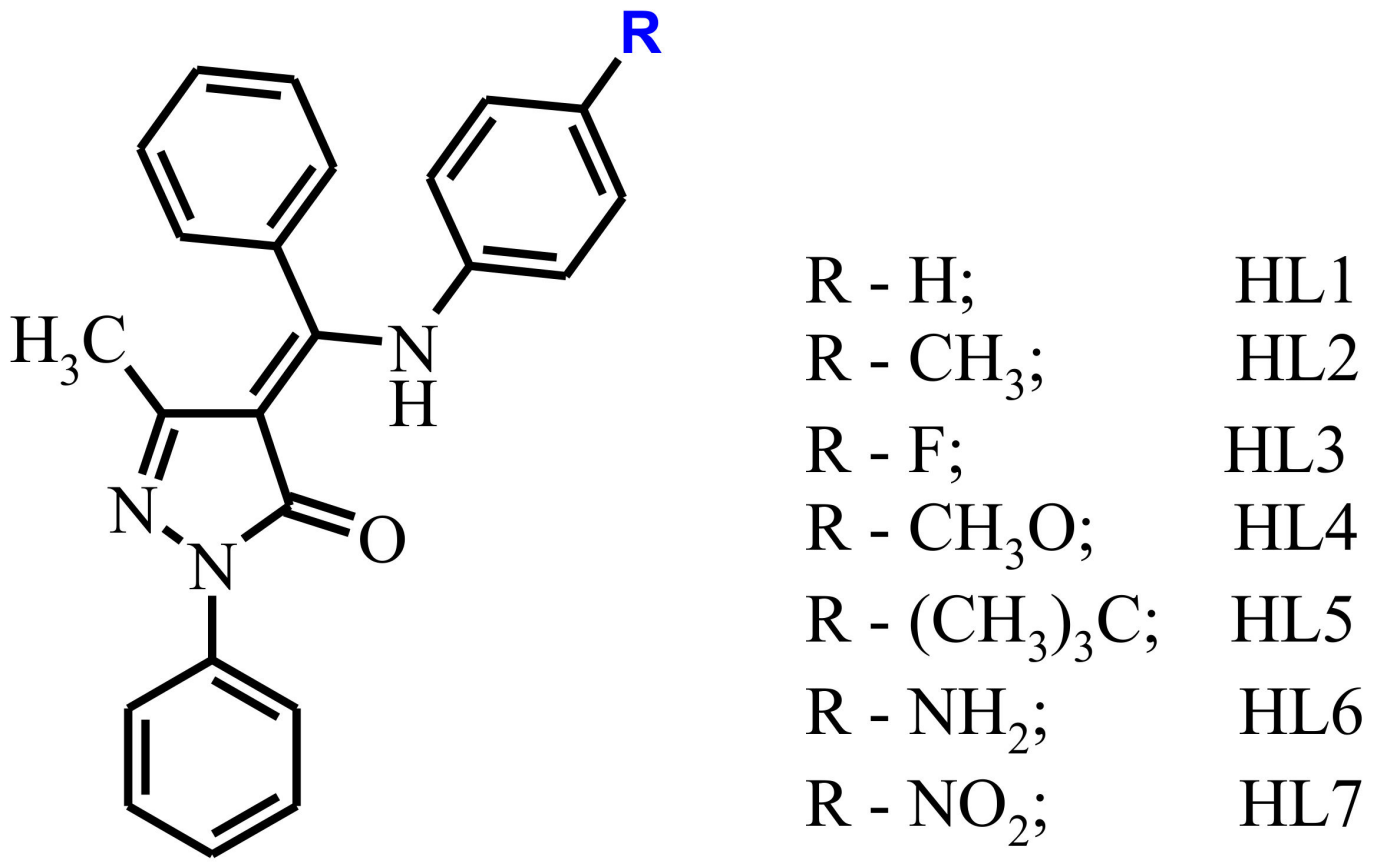
Molecular sketch and designation of the ligands.

In continuation of our studies of the photophysical properties of zinc complexes which contain Schiff-base ligands (Scheme 1) with different substituents in aromatic moiety. The changes of 4-substituent on the aniline unit allow the complexes to retain a luminescent core, and also allow a comparison of the effect of the electronic state.

## 2. Results

### 2.1. General Characterization

All the reported complexes were synthesized by the following sequential route.

**Scheme 2:**
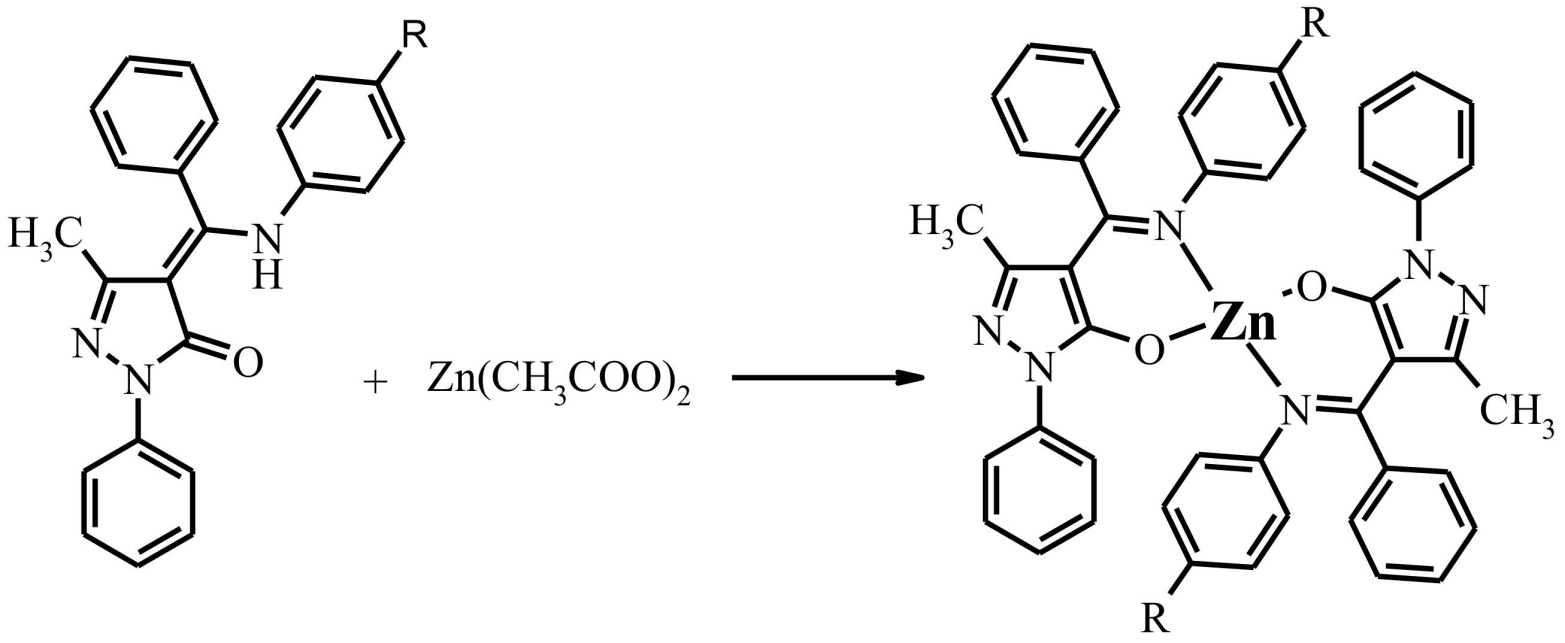
Synthesis of complexed 1–7. Designation of the Zn(L1)_2_-1, Zn(L2)_2_-2, Zn(L3)_2_-3, Zn(L4)_2_-4, Zn(L5)_2_-5, Zn(L6)_2_·EtOH-6, Zn(L7)_2_-7.

The general sketch of title Zn complexes is shown in Scheme 2. The complexes are closely related and were characterized by ^1^H NMR, electrosprey mass spectra (ES-MS), infrared (IR) and Ultraviolet–visible (UV–vis) spectroscopies. According to the element analysis data, these complexes possess Zn:L = 1:2 molar ratio. ES-mass spectroscopic data clearly suggested that mononuclear species had formed in each case. IR, ^1^H NMR reveal that formation of the deprotonated imine-ole form of ligand upon coordination.

Thermal stability is one of the main important parameters of the complexes applied for producing thin-layer luminescent materials by vacuum deposition techniques [22]. The thermogravimetric analysis (TGA) of the complex revealed that there were no solvate molecules present in the lattice of the zinc complexes 1–7. The complexes displayed a melting point at around 225–267 °C and remained stable up to a temperature of 270–315 °C. Further increase in temperature lead to the decomposition of the organic part of molecules 1–5 and 7 in the temperature range of 350–570 °C. Complex 6 thermally decomposed in two steps. The first weight loss of 5.6% at around 90–160 °C corresponds to the release of the lattice ethanol molecule. The desolvation of the title complex leads to stable phases with thermal stability up to 320 °C, followed by a decay of the complexes, via burning of the organic ligand. The process is completed at a temperature of 590 °C.

### 2.2. Structure of Complex 4

**Figure 1:**
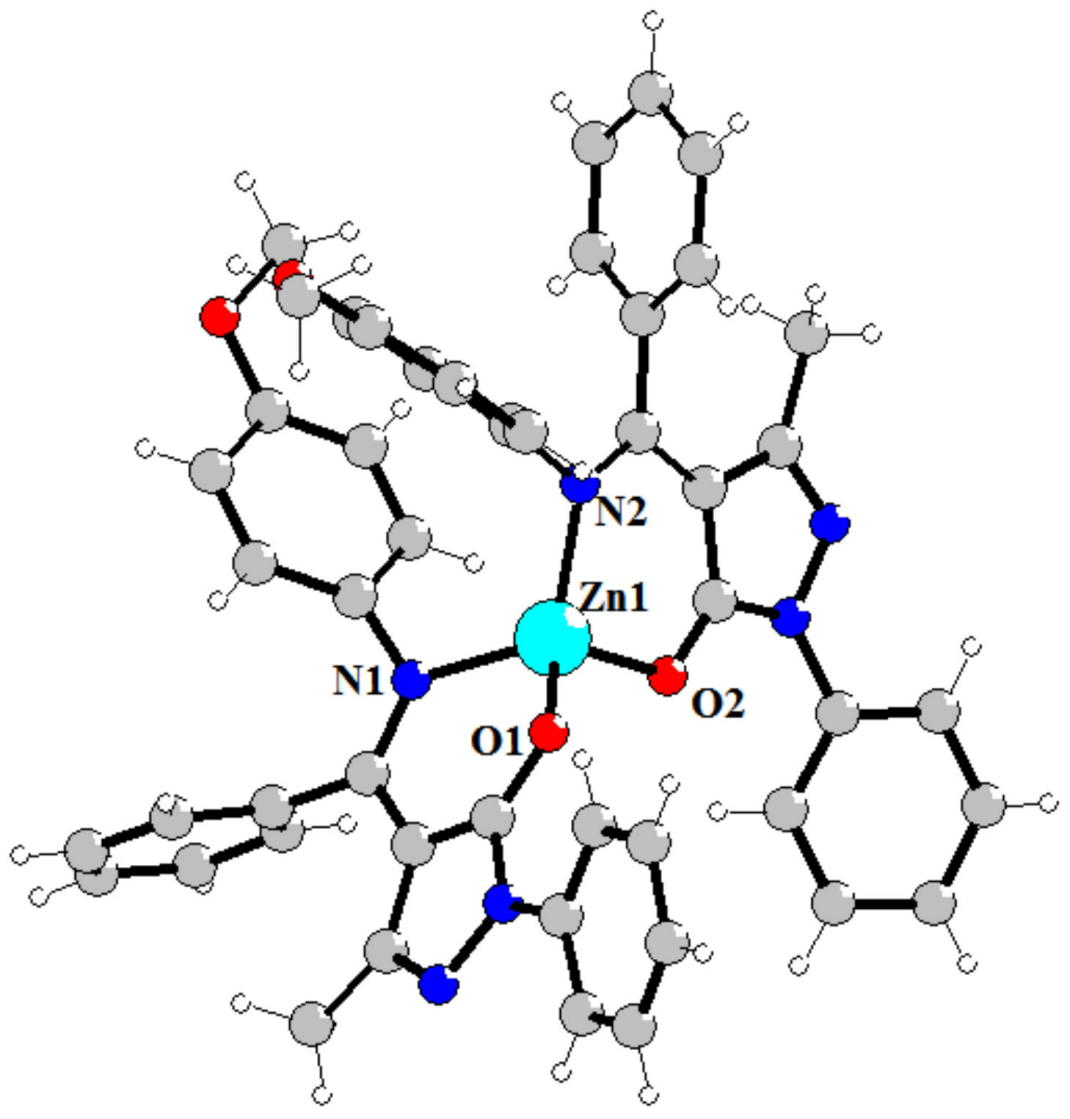
Structure of complex 4. Selected bond’s length (Å): Zn1-O2 1.9542(16), Zn1-O1 1.9437(16), Zn1-N2 1.9788(19), Zn1-N1 2.0089(19), Zn2-O6 1.9501(16), Zn2-O5 1.9584(16), Zn2-N7 2.0049(19), Zn2-N8 1.9887(19).

**Scheme 3:**
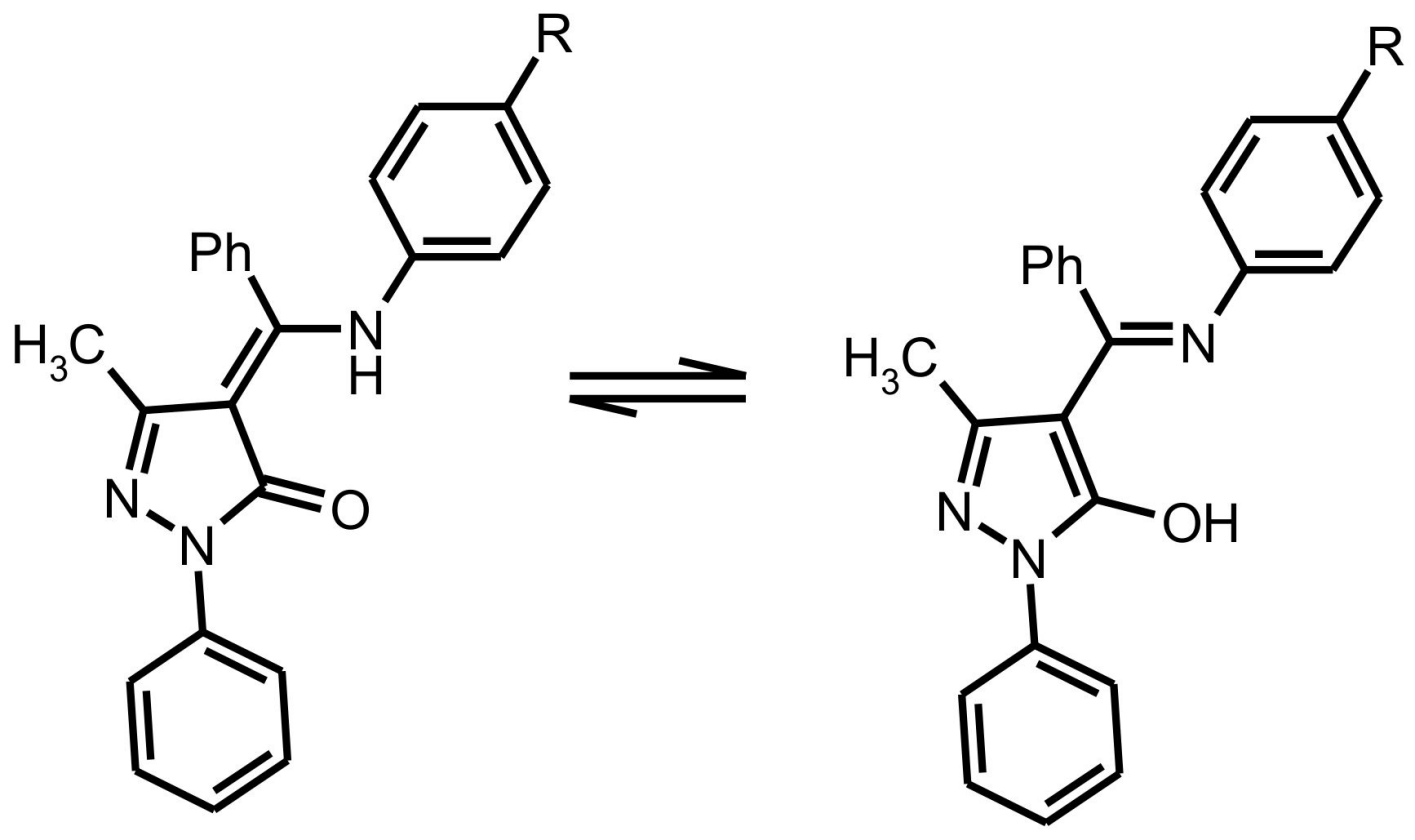
Transitions between tautomeric forms of ligands upon coordination.

The molecular structure of the complex 4 are shown in Figure 1. There are two independent molecules of complex with similar bond’s parameters in the asymmetric unit. Complex 4 consist Zn cation and two L4–anions that make them electroneutral. Careful analysis of N1-C11 and N2-C25 bond’s lengths (1.314 Å) which are close to double C=N length confirms spectral data about coordination of the ligand in the deprotonated imine-ol form (Scheme 3). Both of the two L4 ligands adopt N,O-chelating bidentate coordination mode. The Zn(II) in a four-coordinate environment is composed of two oxygen atoms (Zn–O bonds in the 1.944–1.958 Å range), and two nitrogen atoms (Zn–N bonds in the 1.988–2.009 Å range) from the two L4 ligands. Donor atoms occupy each vertex of the pyramid, forming a slightly distorted tetrahedral geometry. The coordination of the ligands gives rise to a six-membered ring, which is inherently coplanar as a pyrazole ring and indirectly results in the extended conjugate. In both molecules, the bond angle O–Zn–N (95.79–98.96°), formed by oxygen and nitrogen atoms of the same ligand together with the zinc atom, is significantly inferior to the value of the angles (110.63–125.55°) with donor atoms from different molecules, because of the tension of the chelate ring. Chelating and pyrazole cycles are almost co-planar, but both phenyl rings are rotated relative to above cycles.

### 2.3. Photophysical Studies

**Figure 2:**
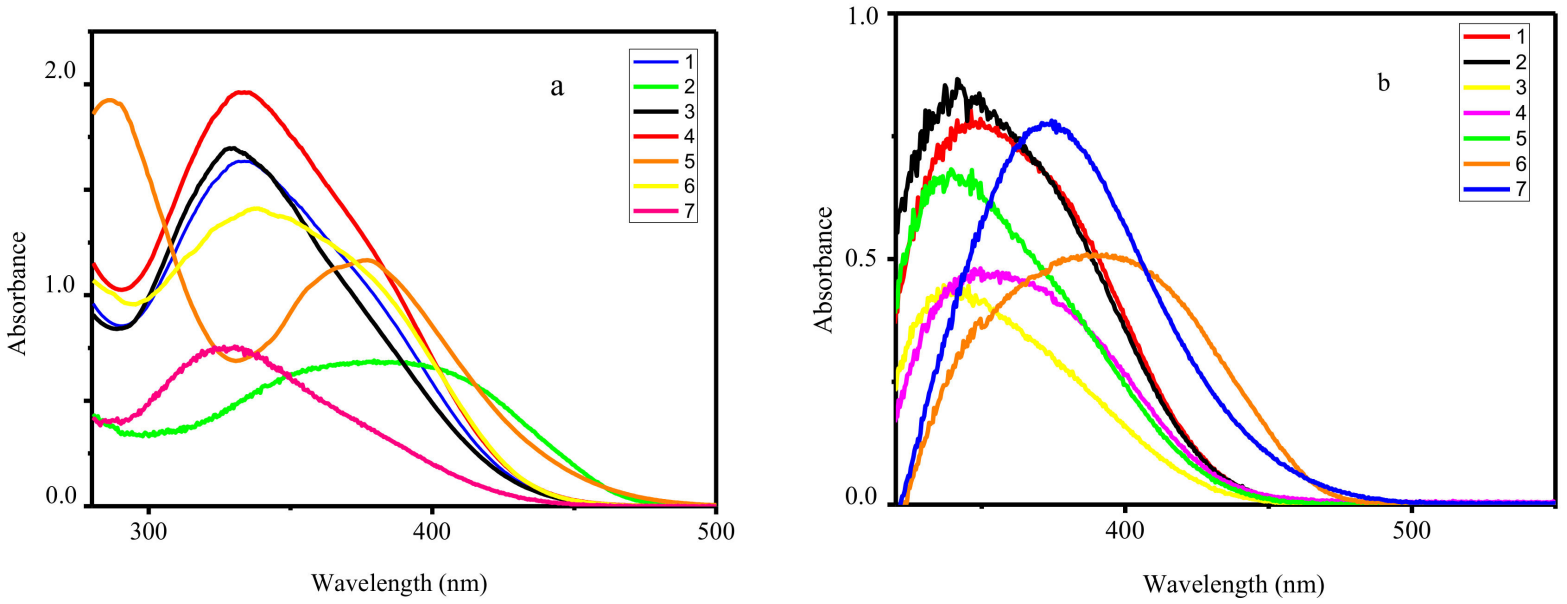
UV−vis absorption spectra of HL1–HL7 (**a**) and corresponding complexes 1–7 (**b**) in CH_3_CN at room temperature.

The UV−vis absorption spectra of the ligands HL1–HL7 and corresponding complexes were measured in CH_3_CN solution (10^−4^ M) at room temperature. The spectra of all Zn complexes exhibit identical profile with one distinct absorbance band centered at 399–424 nm assigned to the metal perturbed intraligand π–π* transition of the C=N azomethine units (Figure 2). Varying of substitution in organic ligands changed the energy difference between highest occupied molecular orbital (HOMO) and lowest unoccupied molecular orbital (LUMO) as results of different electron-donating/withdrawing effect and leading to λ_abs_ shifted. Moreover, coordination of HL1–HL7 leads to the bands shifting to lower energy regions on 7–9 nm, suggests the coordination of azomethine group to zinc cation.

Luminescent properties 1–7 complexes and corresponding ligands were examined in the solid state at room temperature and summarized in Table 1.

**Figure 3:**
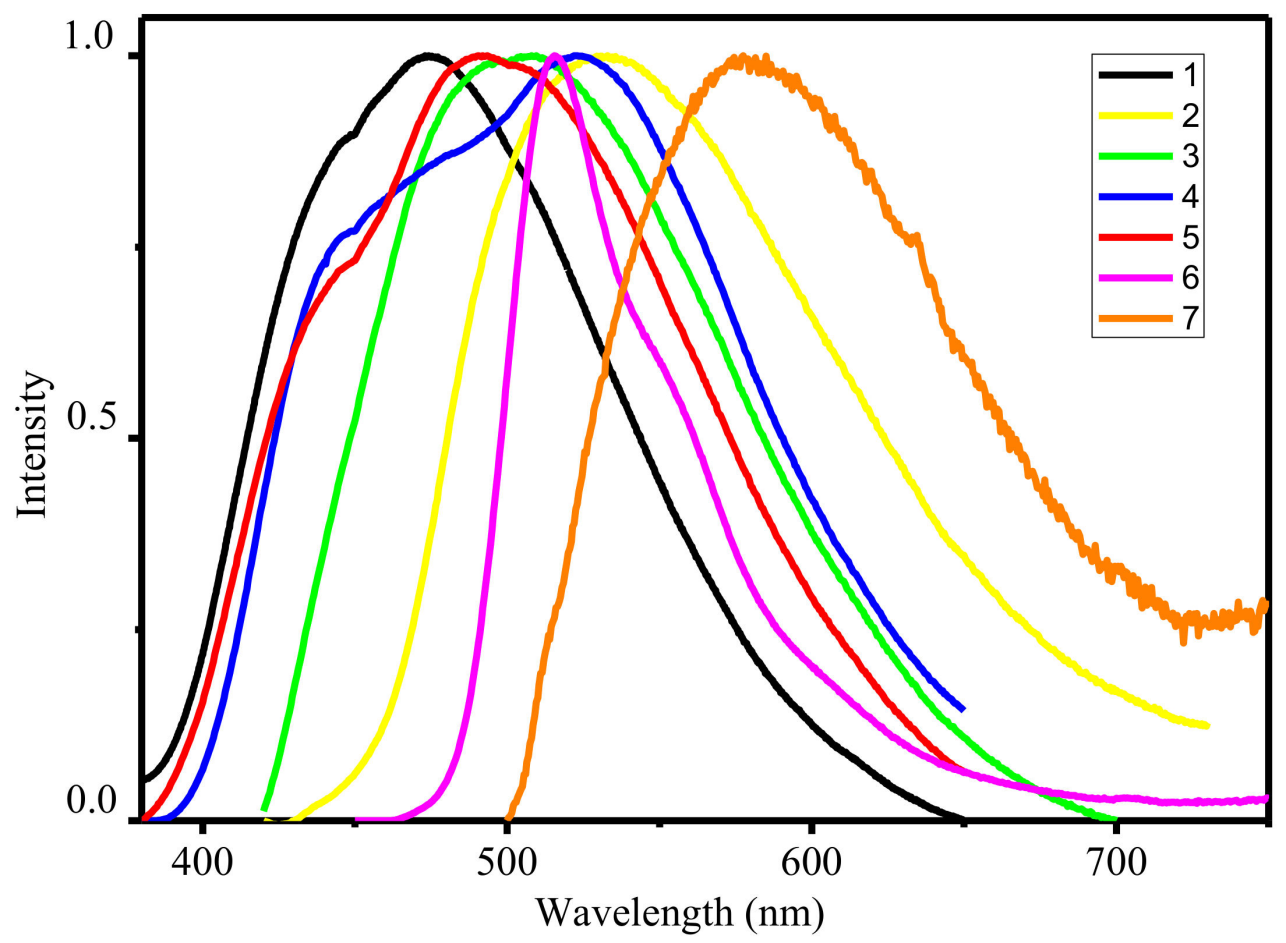
Normalized emission spectra of zinc complexes in solid state.

All complexes in solid state under UV illumination display strong emission in the visible region. The luminescence spectra of 1–7 (see Figure 3) exhibited one broad band with an emission maximum centered at 474–577 nm, assigned to the ligand-centered π*–π transitions. It is well known that the deprotonation of organic ligands and complexation with zinc ions considerably decrease the energy gap between the HOMO and LUMO [22]. In this way, the maximum of emission spectra of the zinc(II) complexes should be bathochromic-shift in relation to the spectra of the corresponding ligands. However, the analysis of the experimental data shows the opposite effect. The reason for the observed phenomenon is associated with tautomeric transitions upon coordination. According previous studies in solid state free ligands exist in amine-one tautomeric form A with a greater degree of conjugation then in imine-ol coordinated form B [23].

Particularly, the highest quantum yields was found for of 4 while lowest—for 7. It is well known that zinc cations in complexes play a key role in increasing the fluorescent emission due to enhancement in the rigidity of the ligand and reduces the loss of energy by thermal vibrational decay, contributing to an increase in the lifetime of the excited state and efficiency of luminescence [12,13,14,15,16,17,18,19,20,21,22]. To support this, the luminescence decay curves for both ligands and their corresponding complexes were recorded at room temperature in the solid state. The detailed data are listed in Table 1. The obtained data clearly indicate an increase in the lifetime upon passing from azomethines to complexes. A general trend that can be observed is that the luminescence lifetimes for complexes 2–7 with substituted ligands are longer than that of complex 1. The most obvious observation is that the lifetime of 1 (τ = 12.8 ns) is 2.9-fold than of complex 7 (τ = 4.75 μs) in solid state. The quantum yields (QY) of all complexes have been determined by absolute method at 298 K (Table 2). It was found that the quantum yields of complexes significantly enhanced with compare with ligand and lies in the range 3.1–49.2% which makes them promising as materials for the emissive layers.

Another interesting result is the ability to fine-tune the fluorescent colour of the complexes by shifting of the emission maximum wavelength due to varying the R group (see Table 2) [24]. The solid-state normalized luminescent spectra of the seven complexes virtually cover the entire visible spectrum. Complex 1 with unsubstituted ligand demonstrate in the solid state a blue luminescence (CIE 0.175; 0.209), which make them promising materials for producing full-colour OLED devices. Emission of complexes with substituted ligands are red-shifted compared to that of 1. The highest red-shifting was found for the complex 7 containing NO_2_ substituent with orange emission (CIE 0.485; 0.505). Obtained luminescent data of zinc complexes 1–7 indicate that the adjustable optical properties can be tuned by relatively minor peripheral variations in ligand structure. It should be noted that such tuning should not strong impact negatively on the quantum yield of emission. Variation of the substituents on the ligand has a relatively minor effect, with the exception of complex 7, which shows a high decrease in emission quantum yield.

### 2.4. Electrical Properties

**Figure 4:**
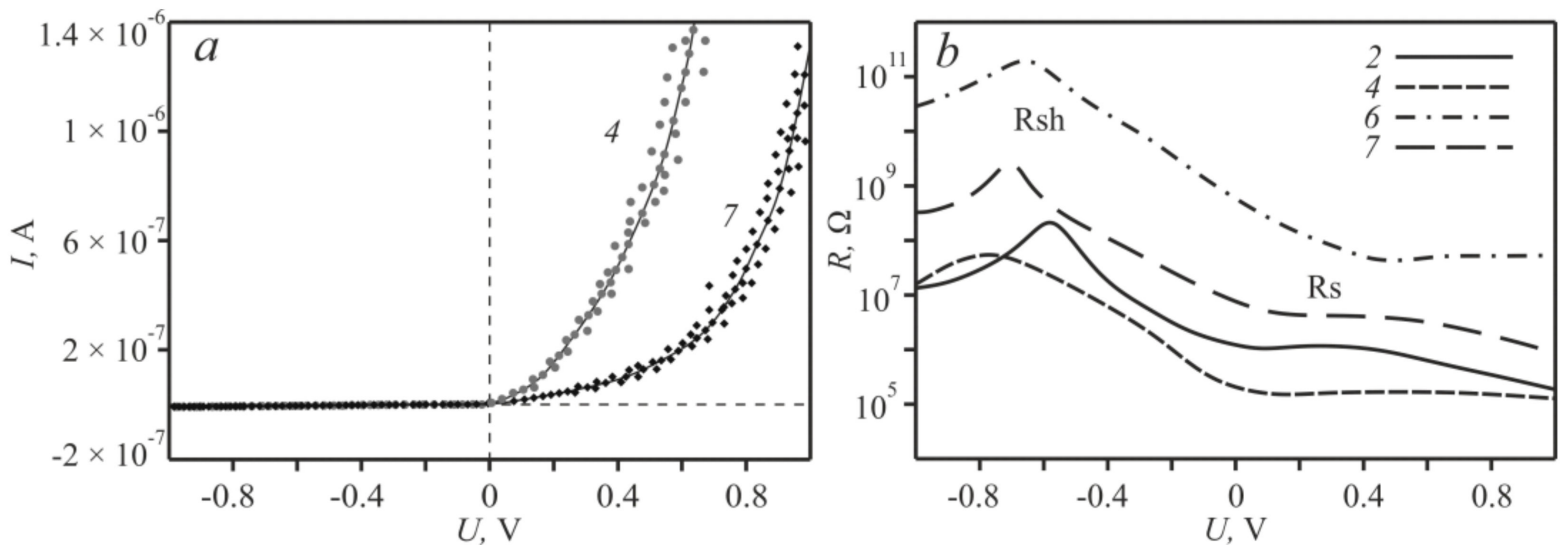
Current–voltage characteristics (**a**); and the dependence of the resistance on the bias voltage of the/Al/Zncomplex/ITO structure (**b**).

For designing of organic heterostructures based [25,26] on the zinc complexes its electrical properties in the ITO-Zncomplex-Al “sandwich” were studied. Of all the above-mentioned modifications of zinc complexes, only based on 2, 4, 6 and 7 structures are showed the presence of diode characteristics. The maximum ratio of the direct current to the reverse current was traced for sample 7 and was determined to be 404 (Figure 4a), while the minimum change in current was found for the sample 2–46 times. The current–voltage curves for structures based on 3 and 5 have a symmetric form. Resistance (Rs) and shunt resistance (Rsh) were also determined for the obtained diodes. Ideally, the series resistance should be zero and the shunt resistance should be about 10^9^ Ω [27]. Therefore, for each of the four variations of the structures with diode characteristics, the logarithmic dependences of the voltage increment on the current increment (*d*V/*d*I) were plotted (Figure 4b). From the obtained curves the operating values of Rs and Rsh were determined. Note that as the bias voltage increases, the resistance approaches a constant value of Rs. With a reverse bias voltage, the resistance Rsh behaves in a similar way, from which the parameters of the series and shunt resistances were obtained (Table 2).

**Figure 5:**
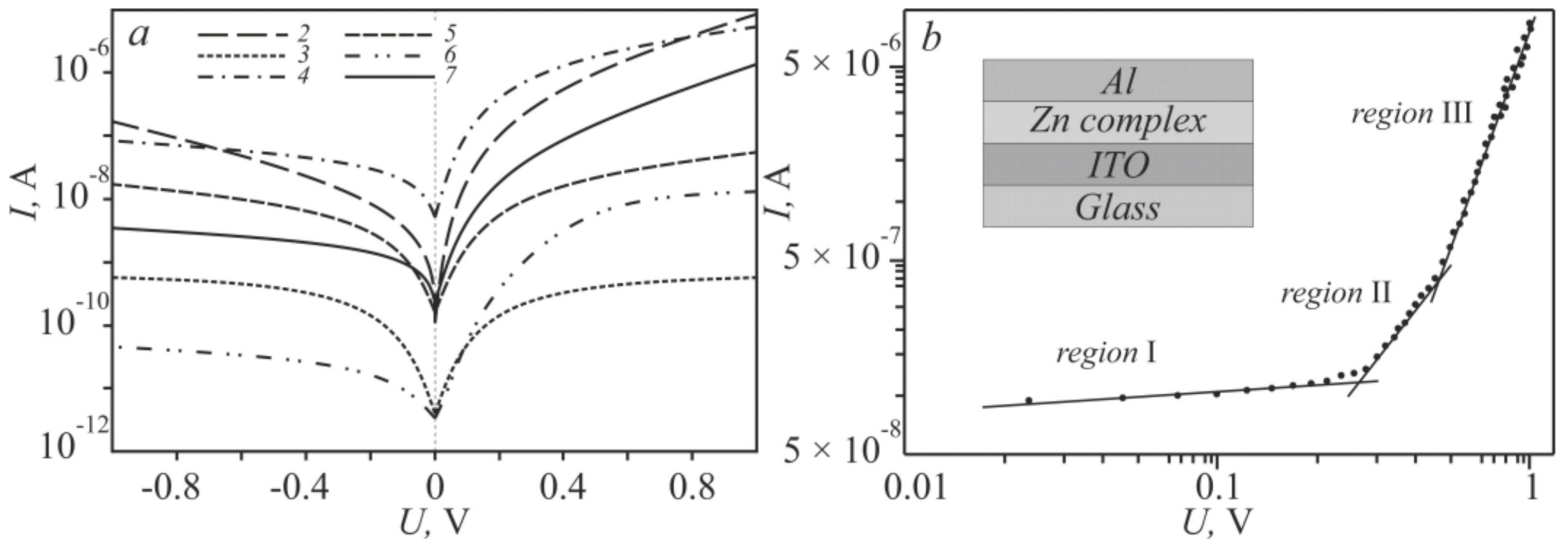
Logarithmic current–voltage characteristics of the zinc complex (**a**); Linearization of the I–V characteristic 2 (**b**).

To estimate the exponential increase in the forward current with the applied voltage and a slow change in the reverse current of the obtained current–voltage characteristics (Figure 4a), the “sandwich” structures (ITO/Zncomplex/Al) were rearranged into a logarithmic scale (Figure 5a), which clearly show typical diode dependences of the Schottky type.

Based on this, a classical model describing the behavior of Schottky diodes can be applied to the obtained I–V characteristics, in which the dependence current vs. applied voltage is determined as [28]:

(1) $$I=I_{0}\left[\exp\left(\frac{qV}{\eta kT}\right)-1\right]$$

where *I*_0_ is the saturation current, *q* is the electron charge, *V* is the applied voltage, *k* is the Boltzmann constant, *T* is the temperature, and *η* is the ideality factor. The saturation current density *I*_0_ can be calculated from the direct intersection of the line ln (*I*_0_) at *V* = 0 [29] which is determined by the equation:

(2) $$I_{0}=AT^{2}\exp\left(\frac{-q\varphi_{b}}{kT}\right)$$

where *A* is Richardson’s constant equal to 120 A cm^−2^ K^−2^ [30], and *φ_b_* is the height of the barrier. The barrier height *φ_b_* can be expressed from Equation (2) as follows:

(3) $$\varphi_{b}=\frac{kT}{q}\ln\left(\frac{AT^{2}}{I_{0}}\right)$$

Using dependence (3), it can be shown that the use of Schiff base ligands with different functional groups made it possible to change the barrier height *φ_b_*. Table 1 shows the barrier heights for four types of zinc complexes.

The ideality factor *η*, which determines the possibility of using the structure in organic electronics, was determined from the slope of the linear part of the forward displacement of the ln(*I*)–ln(*V)* graph (Figure 5b) and was calculated from the relation [31]:

(4) $$\varphi_{b}=\frac{kT}{q}\ln\left(\frac{AT^{2}}{I_{0}}\right)$$

To determine the conduction mechanism in the obtained organic diode structures, their ideality coefficient *η* was compared with the parameters of an ideal diode, the value of which is equal to unity. In this case, the pure thermionic emission mechanism is dominant. The obtained values of *η* for the samples under study are much greater than unity (Table 2), therefore, their conduction mechanism is much more complicated [32]. High values of the ideality factor led to interphase states of the influence of barrier inhomogeneity [33].

To determine the dominant charge, transfer mechanism in the ITO-Zncomplex-Al “sandwich” structure, the characteristics were analyzed on a logarithmic scale (Figure 5b). In this case, the dependence of the current on the applied voltage is described by the power law I~Vm [34], where m is the slope value for each region of the applied voltage, characterizes the kinetics of charge carriers. The resistive mechanism, characterized by the m order of unity (Table 2, the value of m1), for the structures 2 and 7 operates in the interval 0 < V < 0.3 V, for 6–0 < V < 0.15 V, and for 4–0 < V < 0.5 V. The current mode by the m of the order of two and higher (Table 2, the value of m2) is inherent in space charge limited conduction (SCLC) [35] in the experimental structures 6 and 2, which worked at voltages less than 0.6 V, for 7–0.6 V, 4–1 V. Power-law values of m are more than 3 (Table 2, the value of m3), almost all experimental structures had a voltage of ~1 V, and only for 4 did these values exceed 1.5 V. Therefore, the charge transfer is regulated by the mechanism trap charge limited conduction (TCLC) [36].

## 3. Materials and Methods

### 3.1. Materials

All starting reagents and chemicals were purchased from Aldrich (Sigma-Aldrich, St. Louis, MO, USA) and used as received. Starting 1-phenyl-3-methyl-4-benzoyl-5-pyrazolone based ligands were prepared according to the literature methods [16]. Absorption UV–vis spectra were recorded on a Cintra 4040 spectrophotometer (GBC Scientific Equipment Pty Ltd, Braeside, Australia). IR spectra were measured with a FSM 2202 spectrometer (Infraspek, Sankt-Petersburg, Russia) with KBr pellets in the range 400–4000 cm^–1^. ^1^H NMR spectra were recorded in D_6_-DMSO using a Bruker 500 MHz Avance III spectrometer (Bruker Corporation, Billerica, MA, USA). Thermogravimetric experiments were performed on a STA6000 (PerkinElmer, Inc., Shelton, CT, USA) under static nitrogen atmosphere. Luminescence spectra were recorded on a FluoroMax-4 spectrofluorimeter (HORIBA Scientific, Kyoto, Japan). Solid samples quantum yields were determined under ligand excitation using an integrating sphere absolute method. Lifetime measurements were performed on a Horiba Fluorocube lifetime instrument (HORIBA Scientific, Kyoto, Japan) by a time-correlated single-photon counting method using a 365 nm LED excitation source. Elemental analyses of C, H, and N were performed with a EuroEA3000 analyzer (EuroVector, Milan, Italy).

### 3.2. Thin Films Preparations and Studies

To study the conductive properties of zinc complexes, thin films were prepared by pouring from appropriate solutions. Chloroform (CHCl_3_) (Sigma-Aldrich, St. Louis, MO, USA) was used as a solvent. After thorough mixing and holding (for at least 48 h at 293 K), the resulting suspensions were applied to Al and ITO (indium tin oxide), with a volume of the latter of 1 mL. This is due to the fact that the electron work function of ITO is comparable to that of polymers, which provides ohmic contact with the organic layer, while Al forms a rectifying barrier due to the lower work function [37,38]. The contact group was obtained by magnetron sputtering. The ITO resistivity was 16–18 Ω/sq, and aluminum had a resistivity of 20 Ω/sq. The geometric parameters of both constituent contact groups, Al and ITO, were 10 × 10 mm. Electrical studies were performed using a Keysight B1500 semiconductor analyzer (Keysight Technologies Inc., Santa Rosa, CA, USA) in a shielding chamber (Faraday cage). In this case, the maximum voltages in amplitude did not exceed ± 5 V and were limited in current to 100 mA.

### 3.3. Synthesis of Target Complexes

The title Zn complexes were obtained by the following general procedure.

Corresponding ligands (2 mmol) and Zn(AcO)_2_·2H_2_O (219 mg, 1 mmol) were suspended in 50 mL of anhydrous ethanol. The suspension was stirred for 4 h at 60 °C. During that time a light yellow precipitate are formed. After cooling to room temperature precipitate was collected by filtration, washed with cold ethanol and dried in a vacuum.

Zn(L1)_2_ (1), light-yellow solid, yield: 85%. FTIR (KBr, cm^−1^): 1590, 1572, 1526, 1482, 1453, 1376, 1213, 756, 686. Elemental analysis (%) calculated for C_46_H_38_N_6_O_2_Zn (%): C, 71.55; H, 4.96; N, 10.88; Found (%): C, 71.46; H, 4.66; N, 10.80. ESI–MS molecular-ion peak *m*/*z* = 773.21, corresponding to [Zn(L1)_2_ + H^+^]. ^1^H NMR, d (ppm): 1.44 (3H, s, CH3), 6.50–8.01 (15H, m, Ar–H).

Zn(L2)_2_ (2), light-yellow solid, yield: 85%. FTIR (KBr, cm^−1^): 1590, 1573, 1546, 1483, 1456, 1379, 1216, 754, 686. Elemental analysis (%) calculated for C_48_H_42_N_6_O_2_Zn (%): C, 72.04; H, 5.28; N, 10.50; Found (%): C, 72.19; H, 5.72; N, 10.36. ESI–MS molecular-ion peak *m*/*z* = 801.26, corresponding to [Zn(L2)_2_ + H^+^]. ^1^H NMR, d (ppm): 1.42 (3H, s, CH_3_), 2.13 (3H, s, CH_3_), 6.64–8.09 (14H, m, Ar–H).

Zn(L3)_2_ (3), light-yellow solid, yield: 85%. FTIR (KBr, cm^−1^): 1590, 1575, 1527, 1476, 1456, 1376, 1207, 756, 689. Elemental analysis (%) calculated for C_46_H_36_F_2_N_6_O_2_Zn (%): C, 68.36; H, 4.49; N, 10.40; Found (%): C, 68.21; H, 4.24; N, 10.15. ESI–MS molecular-ion peak *m*/*z* = 809.21, corresponding to [Zn(L3)_2_ + H^+^]. ^1^H NMR, d (ppm): 1.47 (3H, s, CH_3_), 6.71–8.16 (14H, m, Ar–H).

Zn(L4)_2_ (4), light-yellow solid, yield: 85%. FTIR (KBr, cm^−1^): 1590, 1573, 1529, 1480, 1456, 1376, 1239, 770, 689. Elemental analysis (%) calculated for C_48_H_42_N_6_O_4_Zn (%): C, 69.27; H, 5.08; N, 10.10; Found (%): C, 69.39; H, 4.82; N, 10.52. ESI–MS molecular-ion peak *m*/*z* = 833.28, corresponding to [Zn(L4)_2_ + H^+^]. 1H NMR, d (ppm): 1.46 (3H, s, CH_3_), 4.26 (3H, s, CH_3_), 6.57–8.05 (14H, m, Ar–H).

Zn(L5)_2_ (5), light-yellow solid, yield: 85%. FTIR (KBr, cm^−1^): 1590, 1575, 1537, 1480, 1454, 1373, 1216, 755, 690. Elemental analysis (%) calculated for C_54_H_54_N_6_O_2_Zn (%): C, 71.55; H, 4.96; N, 10.88; Found (%): C, 71.46; H, 4.66; N, 10.80. ESI–MS molecular-ion peak *m*/*z* = 884.42, corresponding to [Zn(L5)_2_ + H^+^]. ^1^H NMR, d (ppm): 1.41 (3H, s, CH_3_), 2.02 (9H, s, C(CH_3_)_3_), 6.48–8.07 (14H, m, Ar–H).

Zn(L6)_2_·EtOH(6), yellow solid, yield: 85%. FTIR (KBr, cm^−1^): 1608, 1591, 1516, 1474, 1382, 1361, 1223, 1085, 834, 752, 695. Elemental analysis (%) calculated for C_48_H_46_N_8_O_3_Zn (%): C, 67.96; H, 5.46; N, 13.21; Found (%): C, 68.20; H, 5.18; N, 13.04. ESI–MS molecular-ion peak *m*/*z* = 819.25, corresponding to [Zn(L6)_2_ + H^+^]. ^1^H NMR, d (ppm): 1.41 (3H, s, CH_3_), 6.44 (2H, s, NH_2_) 6.87–8.02 (14H, m, Ar–H).

Zn(L7)_2_ (7), yellow solid, yield: 85%. FTIR (KBr, cm^−1^): 1602, 1575, 1519, 1477, 1456, 1377, 1340, 1236, 759, 695. Elemental analysis (%) calculated for C_46_H_36_N_8_O_6_Zn (%): C, 64.07; H, 4.21; N, 12.99; Found (%): C, 63.86; H, 4.03; N, 12.86. ESI–MS molecular-ion peak *m*/*z* = 863.21, corresponding to [Zn(L7)_2_ + H^+^]. ^1^H NMR, d (ppm): 1.47 (3H, s, CH_3_), 6.67–8.11 (14H, m, Ar–H).

### 3.4. X-ray Structural Analysis

Crystallography diffraction intensity data for 4 were collected using the SuperNova diffractometer (Agilent Technologies, Santa Clara, CA, USA) equipped with a HyPix-3000 detector (Rigaku, Tokyo, Japan) and a micro-focus CuKα radiation source (λ = 1.54184 Å) at the Centre for X-ray Diffraction Studies of Research park of St. Petersburg State University. The structures of the complexes were solved by the direct methods and refined in the full-matrix anisotropic approximation for all non-hydrogen atoms. The hydrogen atoms of the water molecule were found in differential Fourier maps and their parameters were refined using the riding model. The hydrogen atoms of the carbon-containing ligand were geometrically positioned and refined by using a riding model. All the calculations were performed by direct methods and using the SHELX–2014 program package [39,40]. Crystallographic details and the structure refinement statistics for 4 at T = 100(2) K are as follows: C_96_H_80_N_12_O_8_Zn_2_, Mw = 1660.46, prismatic yellow crystals, 0.20 × 0.10 × 0.10 mm, space group $P\bar{1}$, a = 13.8473(3), b = 17.0852(3), c = 19.8698(3), α = 113.639(2), β = 91.427(2), γ = 109.666(2), V = 3985.00(15) Å^3^, Z = 2, d_calc_ = 1.384 g cm^−3^, μ = 1.293 mm^−1^, 2.4640 ≤ θ ≤ 76.8900, 16,406 measured with I > 2σ (I), GOOF = 0.943, R_1_ (I > 2σ (I)) = 0.0509, wR_2_ (I > 2σ (I)) = 0.1428, R_1_ (all data) = 0.0556, wR_2_ (all data) = 0.1465. More detail can be found in CCDC 2038692.

## 4. Conclusions

In summary, seven novel Zn(II) complexes based on Schiff-base ligands were successfully prepared and well characterized by spectroscopic, XRD and thermal analysis techniques. The obtained results indicate that the title complexes show strong luminescence in solid state in combination with good thermal properties and can be recommended as emitting materials in electroluminescent diodes. The electrical properties of the complexes under study in the “sandwich” structure showed a change in the shunt resistance Rsh in a fairly wide range from 0.04 to 145 TΩ, while the height of the potential barrier varied from 0.85 to 1.07 eV. The maximum ratio of the forward current to the reverse current was observed in sample 7 and amounted to 404, and the minimum in the 2 sample—46. The current–voltage characteristics of organic materials 5 and 3 were symmetrical. It was shown that almost all conduction mechanisms inherent in classical diodes are observed in ITO/Zncomplex/Al barrier structures.

## Figures and Tables

**Table 1:** UV–vis, photoluminescence spectra data for title ligands and complexes.

Substance	Absorbance λ_max_ (nm)	Emission λ_max_ (nm)	Quantum Yield %	Lifetime ns	CIE Coordinates **
HL1	329	592	2.1	4.1	-
1	338	474	23.6	12.8	0.175; 0.209
HL2	333	604	1.81	4.5	-
2	340	533	11.2	9.0	0.348; 0.530
HL3	326	580	1.2	4.3	-
3	335	495, 508	21.1	9.5	0.240; 0.378
HL4	337	601	2.2	5.1	-
4	346, 358	470 (sh *) 523	49.2	10.8	0.200; 0.304
HL5	338	598	1.6	4.8	-
5	347	490	30.9	10.2	0.204; 0.297
HL6	383	615	1.3	4.0	-
6	390	515	12.2	7.8	0.284; 0.645
HL7	285, 377	621	0.4	4.1	-
7	372	577	3.1	6.5	0.485; 0.505

Sh *—shoulder; CIE Coordinates **—Commission Internationale de l’éclairage.

**Table 2:** Diode parameters of the Al/Zncomplex/ITO (Indium tin oxide).

Diode	Barrier Height Φ_B_ (eV)	Ideality Factor η	Series Resistance Rs (MΩ)	Shunt Resistance Rsh (TΩ)	Slope Value m_1_	Slope Value m_2_	Slope Value m_3_
Al/2/ITO	0.88	3.86	0.62	0.08	0.25	2.24	4.51
Al/4/ITO	0.85	4.39	0.2	0.04	0.56	1.86	-
Al/6/ITO	1.07	2.69	48.9	145	0.95	2.5	4.92
Al/7/ITO	0.95	3.57	3.98	1.76	0.75	2.02	3.91
